# Diurnal Salivary Cortisol in Relation to Body Composition and Heart Rate Variability in Young Adults

**DOI:** 10.3389/fendo.2022.831831

**Published:** 2022-03-09

**Authors:** Selma Cvijetic, Irena Keser, Jasna Jurasović, Tatjana Orct, Željka Babić, Dario Boschiero, Jasminka Z. Ilich

**Affiliations:** ^1^Department of Occupational and Environmental Medicine, Institute for Medical Research and Occupational Health, Zagreb, Croatia; ^2^Laboratory for Nutrition Science, Faculty of Food Technology and Biotechnology, University of Zagreb, Zagreb, Croatia; ^3^Analytical Toxicology and Mineral Metabolism Unit, Institute for Medical Research and Occupational Health, Zagreb, Croatia; ^4^BioTekna^®^ , Venice, Italy; ^5^Institute for Successful Longevity, Florida State University, Tallahassee, FL, United States

**Keywords:** cortisol, heart rate variability (HRV), skeletal muscle, fat mass, bone mass, body composition

## Abstract

**Objective:**

Chronic stress has been related to impaired body composition (bone, muscle, adipose tissue), and disturbed autonomic nervous system (ANS), the latter regulated by hypothalamic–pituitary–adrenal (HPA) axis. Our objective was to investigate the relationship between salivary cortisol, body composition and heart rate variability (HRV—highly influenced by ANS), in a young student population.

**Methods:**

Body composition and HRV parameters were measured using two portable devices based on multi-frequency bioelectrical impedance and photoplethysmography. Body composition included measurement of bone, lean/muscle and adipose tissues. HRV included time domain and frequency domain indices. Salivary cortisol, immediately after awakening, 60 min post awakening and late night was collected and analyzed by ELISA.

**Results:**

Participants included n = 43 healthy university students (39 women, 4 men); 21.1 ± 1.3 years, BMI = 21.8 ± 3.4 kg/m^2^. Skeletal muscle was reduced in most of the participants, bone mass was at the lower normal range, while the fat tissue parameters were normal with only 7% participants being overweight. Cortisol and HRV parameters of sympathetic predominance (low frequency—LF and low frequency/high frequency ratio—LF/HF) were significantly associated with higher fat tissue parameters (fat mass—FM% and intramuscular adipose tissue—IMAT%) (p <0.001 and p = 0.035, respectively) and with lower skeletal muscle (p = 0.002) and bone mass (p = 0.025).

**Conclusions:**

The results point to a role of cortisol and ANS in the control of all three body composition compartments, suggesting that the stress can result in adverse effects on body composition, even in young, apparently healthy individuals.

## Introduction

It is well established that chronic stress could eventually lead to the exhaustion of the hormonal, cardiovascular, neural, and muscular system with pathologic consequences on every organ-system in the body (1, 2), namely, body composition (bone, muscle and adipose tissue impairments) (3); the latter being less studied and understood (4). There are two primary systems that are involved in adapting to the stressful situation: the autonomic nervous system (ANS) and the hypothalamic–pituitary–adrenal (HPA) axis, regulating the former. Among the available biomarkers, heart rate variability (HRV) has been shown as a powerful indicator of the ANS, while salivary cortisol has been suggested as an indicator of the HPA axis functioning (5, 6). Salivary cortisol is still not included in the routine evaluation of cardiovascular or some other chronic diseases risks and the tests require additional validations. Nevertheless, some studies support the use of salivary cortisol as potential diagnostic tools for detecting stress-induced cardiac diseases or to describe the mechanisms by which stress potentially contributes to the pathogenesis and outcomes of cardiovascular diseases or other chronic diseases (7).

Cortisol is secreted by the HPA axis system as a result of both acute and chronic stress response. However, chronic stress could result in the flat-high diurnal secretion (with lower-than-normal level in the morning and higher-than-normal levels in the evening) or flat-low secretion, both resulting in abnormal levels (8). A stress-related elevated cortisol might lead to HPA axis dysregulation, having both psychological and physiological impacts. In terms of physiological impact on body composition, there is a strong relationship between elevated cortisol levels and abdominal obesity, loss of bone mineral density (9), and to the lesser extent, impairments in lean/muscle tissue. Changes in body composition in the state of hypercortisolism are similar to the changes during aging, such as increased overall and visceral fat, decreased lean body mass and bone mineral density (10). Likewise, elevated cortisol has been related to impaired body composition (bone, muscle, adipose tissue) and possibly onset of osteosarcopenic adiposity syndrome (OSA) (4). OSA presents the most advanced stage of body composition deterioration with simultaneous impairment of bone (osteopenia/osteoporosis), muscle (sarcopenia), and excess adipose tissue (11, 12).

Our objective was to investigate the relationship between salivary cortisol, body composition and selected parameters of ANS in the young, healthy, university student population. We wanted to investigate the interaction of body composition with the ANS and HPA system; specifically, whether variation in body composition was accompanied by changes in cortisol secretion and HRV parameters. It is expected that the body composition in young healthy individuals is within a relatively normal range and that diurnal cortisol and HRV parameters follow the regular patterns. The hypothesis was that the possible deviations in body composition parameters are accompanied with aberrant changes in cortisol secretion and HRV and the latter two could easily point to subtle body composition faults.

## Methods

This pilot study included 43 healthy participants (39 women, 4 men), who have been recruited on a voluntary basis at the University of Zagreb. The exclusion criteria were presence of any medical conditions, namely, heart disease, metabolic and endocrine disorders, and also mental disorders. The participants could not be taking any prescribed medications. The questionnaire about overall health status was completed by all and only those who fit the inclusion/exclusion criteria were enrolled in the study; thus, no one was excluded during the duration of the project. Body height (cm) and weight (kg) were measured with standard methods and body mass index (BMI; kg/m^2^) was calculated.

### Bioimpedance Measurements

Two portable devices based on multi-frequency bioelectrical impedance used in the study were BIA-ACC and PPG-StressFlow HRV photoplethysmography (BioTekna^®^, Marcon-Venice, Italy). The BIA-ACC device uses algorithms to provide quantitative and qualitative assessment of body composition: body mass index (BMI; kg/cm^2^), fat mass (FM; kg) also expressed as % of total body weight (TBW; kg), abdominal adipose tissue (AAT; cm^2^), intramuscular adipose tissue (IMAT; as %TBW), fat free mass (FFM; kg and as %/TBW), skeletal muscle (SM; kg) skeletal mass (as %FFM), S-Score (standard deviation of skeletal muscle mass with respect to healthy reference individuals between 25 and 30 years old), skeletal muscle index (wSMI%) calculated as SM/body weight × 100, total bone mass (Bm; kg), and T-Score (standard deviations of the mean bone mass with respect to young healthy reference). The measurements were performed according to the manufacturer’s specifications.

The PPG-StressFlow HRV photoplethysmography device determines a number of parameters to evaluate the physiological activity of the autonomic nervous system (ANS) and HRV and includes time-domain and frequency-domain indices. Cardiac work is influenced by the action of sympathetic (SNS) and parasympathetic (PNS) components of the ANS and it is generally accepted that cardiac “RR intervals” are indicators of ANS activity. RR intervals represent the time in milliseconds between two heartbeats or more precisely between successive R spikes on the electrocardiogram (13). HRV is the measure of the gaps between RR intervals and is used as an index of the influence of both SNS and PNS on cardiac work. The greater HRV is associated with reduced morbidity and mortality, and improved psychological well-being (14). The linear parameters of cardiac action are divided into measures of time and frequency. *Time-domain* indices of HRV quantify the amount of variability in measurements of the inter-beat interval and they include: standard deviation of the heart beat-to-beat interval (SDNN), signifying the overall health of ANS, and root mean square of the differences between adjacent heart beat intervals (RMSSD) expressing the vagal component of parasympathetic nervous system (PNS). Time parameters can only partially describe the functioning of the ANS because they represent the combined action of both subcomponents. In order to separately detect the sympathetic and parasympathetic effects on the heart, it is necessary to apply the procedure of frequency and spectral analysis (15). Accordingly, the sympathetic activity is reflected in the low frequency range of spectral analysis, (0.04–0.15 Hz), because the effects of the SNS on cardiac activity are slow, On the contrary, due to its high speed of action, parasympathetic activity is manifested in the area of higher frequencies of spectral analysis (0.15–0.5 Hz) (16). Therefore, *Frequency-domain* indices are: high frequencies (HF) power (0.15–0.5 Hz), low frequencies (LF) power (0.05–0.14 Hz). The LF reflects the dominance of both SNS and PNS and the HF reflects the dominance of the PNS, while the LF/HF ratio is considered to be an index of sympathetic-parasympathetic balance of cardiac work (17). To obtain ANS tests, participant was sitting in the relaxed position, with two sensors attached on the tips of the second fingers of the hands. These measurements, lasting 5 min, were performed in the morning and in the fasting condition.

### Salivary Cortisol

Participants were individually instructed and provided with saliva-sample collection kits Salivette^®^ Cortisol (Sarstedt, Nümbrecht, Germany) that included synthetic swab designed for cortisol determination and written information about the saliva sampling procedure. The participants were asked not to eat, drink or brush teeth 30 min before sampling. Saliva was collected in the morning after awakening (cortisol 1), 60 min post-awaking (cortisol 2), and before going to bed (cortisol 3) to assess the cortisol awakening response and the slope of cortisol secretion levels during the day. Saliva was obtained as follows: the swab from the Salivette^®^ was removed and placed in the mouth, chewed for about 60 s to stimulate salivation and returned with the absorbed saliva to the Salivette^®^ that was stored at +4°C until delivery to the laboratory. Salivette^®^ were centrifuged at 1,000*g* for 2 min (Hettich Rotanta/R, type 3501, Hettich-Zentrifugen, Tuttlingen, Denmark), the swab removed and clear saliva samples in conical tubes kept frozen at −20°C, for 5–9 months, until analysis.

Salivary free cortisol levels (μg/dl) were measured on a TECAN Infinite M2000 Pro microplate reader (TECAN Group, Männedorf, Switzerland) using the commercial Cortisol Saliva ELISA Kit for the quantitative determination of free cortisol in human saliva (RE52611; IBL International GMBH, Hamburg, Germany) and cortisol concentrations were calculated using a 4-Parameter Logistics curve fit.

### Data Analysis

The results are shown as mean ± standard deviation for continuous variables and as percentages for categorical variables. The distribution of variables was tested using the Kolmogorov–Smirnov test. Almost all variables were normally distributed, but the nonparametric method for testing the differences between groups (Mann–Whitney U test) was used due to small number of participants in some groups. For example, there were only a few students with increased cortisol levels or decreased cortisol 2 level. U test is computed based on rank sums rather than means.

The multiple regression models were created to analyze separately the associations between body composition parameters and cortisol and the associations between body composition and HRV parameters. In the first model, dependent variables were cortisol (morning and evening samples) and in the second model, HRV frequency and time domain parameters (LF, LF/HF, SDNN, RMSSD). Predictors in both models were muscle tissue, fat tissue and bone mass parameters. The calculations were done with the Statistica, 13.0 (Dell Inc., Tulsa, OK). The level of significance was set at p <0.05.

## Results

Mean ± SD age of the participants was 21.1 ± 1.3 years, with mean BMI of 21.8 ± 3.4 kg/m^2^. According to BMI, only 3 women (7%) were overweight and 4 (9.3%) were underweight. Most of the muscle tissue parameters (SM%, wSMI, FFM%) were below the reference values, except SM% in men, which was normal (**Table 1**). Fat tissue parameters (FM%, AAT, IMAT%) were within the reference values, with the exception of FM% in men, which was slightly increased. Bone mass and T-scores were also within the reference values, although the females had bone mass in the lower reference range.

**Table 1:** Age, body composition parameters and salivary cortisol in study participants (N = 43).

Parameter	Mean ± SD	Minimum	Maximum	Ref. range
Age (yrs.)	21.1 ± 1.3	20.0	25.0	
BMI (kg/m^2^)	21.8 ± 3.4	17.3	32.2	18.0–25.0
FFM% (% of BW)	74.9 ± 7.3	50.8	87.0	>75
SM (% of FFM)	30.3 ± 2.5 W	25.7	35.8	>35 W
	42.5 ± 2.9 M	39.6	46.6	>40 M
wSMI	22.8 ± 1.5 W	21.0	28.0	>25 W
	31.0 ± 1.4 M	29.0	32.0	>34 M
S score	−0.6 ± 0.9	−2.2	2.3	>−1.0
FM% (% of BW)	24.3 ± 6.3 W	13.0	40.0	12–30% W
	26.7 ± 6.9 M	22.0	37.0	7–25% M
AAT (cm^2^)	224.9 ± 108.3 W	77.2	595.4	<460 W
	367.0 ± 210.8 M	216.6	678.6	<560 M
IMAT% (% of BW)	1.1 ± 0.4	0.2	2.3	<2.0
Bone mass (kg)	3.0 ± 0.3 W	2.5	4.0	>3.0 W
	4.8 ± 0.7 M	4.1	5.8	>3.9 M
T score	−0.8 ± 0.6	−1.9	1.2	>−1.0
Cortisol 1 (µg/dl)	0.396 ± 0.232	0.135	1.211	0.100–0.750
Cortisol 2 (µg/dl)	0.478 ± 0.354	0.079	1.622	0.100–0.750
Cortisol 3 (µg/dl)	0.039 ± 0.026	0.011	0.119	<0.100

BMI, body mass index; BW, body weight; FFM, fat free mass; SM, skeletal muscle; wSMI, weight adjusted skeletal muscle index; FM, fat mass; AAT, abdominal adipose tissue; IMAT, intramuscular adipose tissue; M, men; W, women.

Source of the reference data for cortisol: Mayo Clinic Laboratories. Rochester 2022 Interpretive Handbook (https://www.mayocliniclabs.com/test-catalog/pod/MayoTestCatalog-Rochester–SortedByTestName-duplex-interpretive.pdf).

Mean values of all cortisol samples were within the reference values (**Table 1**). Two participants (4.6%) had increased cortisol 1, six participants (13.9%) had increased cortisol 2 and only one had increased cortisol 3. Only two participants (4.6%) had decreased cortisol 2. Mean times of cortisol sampling were: 8.3 ± 1.4 hours for cortisol 1; 9.8 ± 1.5 h for cortisol 2; and 23.0 ± 1.0 ho for cortisol 3. All HRV parameters were within the reference values, although the frequency domain parameters were at the lower limit of normal values (**Table 2**).

**Table 2:** Heart rate variability (HRV) assessment in participants (N = 43).

Parameter	Mean ± SD	Minimum	Maximum	Ref. values
HR (bpm)	78.6 ± 12.2	57.8	103.0	46.9–84.8
*Time domain*				
SDNN (ms)	57.6 ± 17.1	24.0	115.0	>50
RMSSD (ms)	42.3 ± 20.8	13.0	116.0	>30
*Frequency domain*				
Total power (Hz)	8.0 ± 0.6	6.3	9.4	≥8
LF (Hz)	6.9 ± 0.6	5.0	8.5	≥6.7
HF (Hz)	6.6 ± 0.9	4.5	8.8	≥6.5
LF/HF	1.7 ± 1.6	0.2	9.0	

HR, heart rate; SDNN, standard deviation of the heart beats interval; RMSSD, root mean square of the differences between adjacent heart beat intervals; LF, low frequency; HF, high frequency.

In the analysis of the relationship between cortisol levels and body composition, the results showed that LF/HF was significantly lower in participants with decreased SM% compared to those with normal SM% (p = 0.048) (**Table 3**). Cortisol levels did not significantly differ between participants with normal or decreased skeletal muscle mass. The same analyses were performed with fat and bone mass and there was no significant difference in cortisol and HRV in participants with normal or higher fat mass. Participants with decreased Bm had significantly lower LF compared to those with normal Bm (p = 0.036).

**Table 3:** Differences in cortisol and HRV parameters according body composition status.

Parameter	SM%		FM%		Bone mass	
	Decreased vs. normal		Increased vs. normal		Decreased vs. normal	
	Z	p	Z	p	Z	p
Cortisol 1	−0.473	0.645	1.763	0.077	−0.393	0.693
Cortisol 2	−1.117	0.272	1.107	0.267	0.063	0.949
Cortisol 3	0.587	0.568	−0.702	0.482	−0.190	0.848
SDNN	−0.890	0.384	0.187	0.851	0.813	0.416
RMSSD	0.000	0.985	0.000	1.000	1.029	0.303
LF	−1.534	0.127	0.530	0.595	**2.096**	**0.036**
HF	0.511	0.619	−0.327	0.743	1.258	0.208
LF/HF	**−1.932**	**0.048**	0.920	0.357	0.304	0.760

SM, skeletal muscle; FM, fat mass; SDNN, standard deviation of the heart beats interval; RMSSD, root mean square of the differences between adjacent heart beat intervals; LF, low frequency; HF, high frequency.

The differences were tested with Mann–Whitney U test, which is accompanied by a Z value (normal distribution variate value) and the respective p-value. Significant values are marked in bold.

Association between cortisol and body composition, and also between HRV and body composition were tested in multiple regression models, controlling for BMI (**Table 4**). First model included cortisol as dependent variable; there was a significant association between cortisol 3 and FM% (b = 0.007, p <0.001) and significant negative association between cortisol 3 and SM% (b = −0.021, p = 0.002). Cortisol 1 and cortisol 2 were not significantly associated with any of body composition variables. In the second model, HRV parameters were dependent variables. The results showed significant positive association between LF/HF and IMAT% (b = 2.40; p = 0.035). Significant predictors of LF were IMAT% (b = 1.07; p = 0.022); SM% (b = 0.48; p = 0.007) and bone mass (b = −3.03; p = 0.025). HF, SDNN and RMSSD were not significantly associated with any of the body composition variables.

**Table 4:** Association between cortisol, LF/HF and LF as dependent variables with body composition parameters.

Parameter	Cortisol 3		LF/HF		LF	
	b	p	b	p	b	p
BMI	−0.001	0.898	1.27	0.108	−0.38	0.176
SM%	**−0.021**	**0.002**	−1.19	0.120	0.08	0.070
S score	0.052	0.056	−2.68	0.125	0.01	0.982
FM%	**0.007**	**0.000**	0.01	0.919	−0.03	0.210
AAT	0.000	0.053	−0.01	0.423	0.00	0.085
IMAT%	−0.008	0.616	**2.40**	**0.035**	**1.07**	**0.022**
Bone mass	0.050	0.500	2.18	0.499	**−3.03**	**0.025**
T score	−0.047	0.092	0.98	0.566	0.49	0.486

BMI, body mass index; SM, skeletal muscle; FM, fat mass; AAT, abdominal adipose tissue; IMAT, intramuscular adipose tissue; LF, low frequency; HF, high frequency.

The associations were tested with multiple regression (b, coefficient of regression). Significant values are marked in bold.

## Discussion

The participants enrolled in our study presented with normal mean values of all three cortisol samples, although 2.9 to 13.3% of participants had elevated levels at some sampling times. Increased evening cortisol was associated with higher fat mass and lower skeletal muscles. Parameters of sympathetic predominance, like LH, were also associated with higher fat tissue and lower bone mass, suggesting that stress indicators are associated with adverse effects on body composition.

To our knowledge, this is the only study to analyze the association of salivary cortisol and HRV parameters with all three components of body composition: body fat, skeletal muscle, and bone tissue. Our study also included a young healthy population, while most other studies were focused on individuals with specific diseases (18, 19), especially with psychological problems (20, 21), athletes, or those involved in some kind of exercise (22, 23).

Surprisingly, our participants had lower skeletal muscle parameters than expected for their young age. Although we did not assess physical activity, we speculate that the level of physical activity was low in this student population due to school obligations and sedentary lifestyle. In our earlier research we found that female students from the University of Zagreb had low to medium level of physical activity, ranging from low to very high (24). In the present study, lower skeletal mass was associated with lower sympathetic–parasympathetic balance (LF/HF) and lower evening cortisol. Similarly, in the regression analysis, lower SM% was associated with higher evening cortisol. It has been shown that a chronically high cortisol level may induce muscle atrophy (25), especially in older population and is also associated with a lower handgrip strength and sarcopenia (26). Although our participants were young with average normal cortisol secretion, some temporary disturbance in diurnal variation of hormone secretion could be stress-induced. However, it is probably reversible at their age and not deemed to produce any lasting effects. The study group had only 7% overweight females. Fat tissue parameters were significantly positively associated with cortisol 3 and also with LF, and LF/HF, reflecting sympathetic predominance in association with total fat tissue and also with impaired adipose tissue distribution. Investigators reported different patterns of autonomic dysfunction in individuals with total or visceral obesity, reflecting either higher or lower sympathetic activity or even a global reduction of cardiac autonomic nervous activity (27). Our results indicate an adverse effect of body fat on sympatho-vagal balance and the shift towards the sympathetic component. Chen et al. (28) also confirmed that the body weight and height were positively correlated with LF/HF. A study conducted by Shetty et al. (29) demonstrated elevated sympatho-vagal balance and reduced parasympathetic control in women with increased BMI. The evidence also showed that the changes in HRV are reversible; reduction of body fat led to improved ANS function.

The participants with lower skeletal mass also had a lower LF/HF indicating parasympathetic dominance. This typically occurs when body conserves energy, but may also reflect muscle fatigue. LF power reflects cardiac sympathetic activity (15), thus, the lower LF can be interpreted as a decrease in the portion of sympathetic HR modulation (13–17). Cardiovascular system responds to muscle action with increase in HR and systolic blood pressure (30), indicating an increased sympathetic drive to the vessels, which should be reflected by an increased LF. Billman et al. (31), proposed that the LF power is not a pure index of SNS drive, but that the half of the variability in this frequency band is due to the PNS and that the LF/HF ratio measures sympatho-vagal balance. Therefore, in our participants low LF/HF ratio could be an indicator of low muscle mass and potential muscle fatigue.

Cortisol acts on bone directly by reducing bone apposition and increasing bone resorption, and indirectly by blocking calcium absorption, leading to disturbance in bone metabolism (32). Our participants had on average normal bone mass, although the mean T-score of −0.7 indicated that the bone mass was not as good as would be expected for a young healthy population. Although a short bout of elevated cortisol secretion related to stress may cause a decrease in BMD (33), we presume that the probable lack of physical activity could have been a predictor of slightly lower bone mass and skeletal muscle mass in these young participants. Bone mass in our participants correlated negatively with LF and LF/HF, pointing to the role of ANS in controlling the musculoskeletal metabolism and to complex interaction between ANS and three body composition compartments (34).

Salivary cortisol measurements have greatly advanced the research of the HPA axis and its regulation of ANS. However, this method has its shortcomings due to different reasons, namely, some of the following: variation in saliva sample collection, participant noncompliance for timing of collection, the number of samplings, and various analytic methodology approaches (35). Nevertheless, it has been demonstrated that salivary cortisol concentrations correlated well with measured serum free cortisol levels, rendering a reliable estimates of serum free cortisol (36). Moreover, measurements of the salivary cortisol have been shown to offer some advantages over serum cortisol, since most of the analyte is in the free form and not bound to corticosteroid binding globulin. Additionally, the sampling is easy and noninvasive, simplifying the ethical and patient-burden issues. The most common inconsistency among different studies is the frequency of saliva sampling. Some studies showed that frequent (10 min) sampling is necessary to fully characterize the pulsatile nature of plasma cortisol (37), while others pointed out that 8-hourly salivary cortisol measurements provide a reliable method of estimating 24-hour cortisol exposure for population studies (38). We have followed recommendations by Adam and Cumari (39) regarding the design of sample collection to assess salivary cortisol in epidemiological research, applying a “minimal protocol”, namely, 3 data points (one sample on waking, one 30 min after waking, and one at bedtime), enabling information on the cortisol awakening response and the cortisol secretion diurnal decline slope.

We are aware that sampling more times a day may be the better indicator of the circadian rhythm of cortisol secretion, but as good correlations between more intensive sampling and the values obtained using “minimal protocols” were shown (39), we believe are reasonable substitute for estimation of cortisol secretion in young healthy individuals.

### Limitations

The limitation of the study includes a relatively small number of participants, reducing the power analysis. However, the participants were a homogeneous group with respect to age and lifestyle since all were university students. Moreover, there are a number of other studies with cortisol investigations, comprising similar or even smaller number of participants but rendering useful results and information (18, 40–44).

Another limitation is the lack of lifestyle data, namely, physical activity, diet, smoking, and the level of psychological stress which all can be confounders of cortisol secretion and HRV. However, based on our previous research and also as evident from other studies, there is certain level of similarity in the population lifestyle of students. It was also found that leisure-time physical activity is below recommended levels in a substantial proportion of European students, and the exam-related stress (24, 45, 46). Based on those findings, it could be assumed that lifestyle characteristics in our study group are similar to published studies of other European students.

## Conclusions

Our results show that in this population of young and healthy college students, average salivary cortisol secretion showed normal diurnal variability in most of the cases. Additionally, we found significant association of higher salivary cortisol with lower skeletal muscle mass and higher fat mass, simultaneously paralleled with the corresponding HRV parameters. These results indicated that both salivary cortisol and HRV parameters are sensitive indicators of changes in body composition. The implications of our results are important in view that salivary cortisol (relatively easily obtained and measured) can be used to assess the psychological and physiological health of young adults, and specifically subtle impairments in body composition, an extremely important component of the health and image of the young population. Additionally, this is the only study that included a large number of body composition parameters and analyzed them in association with diurnal cortisol rhythm and HRV variables in young and healthy population of university students.

## Data Availability Statement

The raw data supporting the conclusions of this article will be made available by the authors, without undue reservation.

## Ethics Statement

The studies involving human participants were reviewed and approved by the Ethics Committee of the Institute for Medical Research and Occupational Health. The patients/participants provided their written informed consent to participate in this study.

## Author Contributions

SC and IK designed the study, conducted the study, performed data analyses and wrote the manuscript. JJ and TO performed laboratory analysis. ZB conducted the study and performed data analyses. DB and JZI reviewed and edited the manuscript. All authors listed have made a substantial, direct, and intellectual contribution to the work and approved it for publication.

## Conflict of Interest

The authors declare that the research was conducted in the absence of any commercial or financial relationships that could be construed as a potential conflict of interest.

## Publisher’s Note

All claims expressed in this article are solely those of the authors and do not necessarily represent those of their affiliated organizations, or those of the publisher, the editors and the reviewers. Any product that may be evaluated in this article, or claim that may be made by its manufacturer, is not guaranteed or endorsed by the publisher.
